# Maximum-Entropy Inference with a Programmable Annealer

**DOI:** 10.1038/srep22318

**Published:** 2016-03-03

**Authors:** Nicholas Chancellor, Szilard Szoke, Walter Vinci, Gabriel Aeppli, Paul A. Warburton

**Affiliations:** 1 London Centre For Nanotechnology 19 Gordon St, London, WC1H 0AH, UK; 2 Department of Electronic and Electrical Engineering, UCL, Torrington Place, London, WC1E 7JE, UK; 3 University of Southern California Department of Electrical Engineering 825 Bloom, Walk Los Angeles CA, 90089, USA; 4 University of Southern California Center for Quantum Information Science Technology 825 Bloom Walk, Los Angeles CA, 90089, USA; 5 Department of Physics, ETH Zürich, Zürich, CH-8093, Switzerland; 6 Department of Physics, École Polytechnique Fédérale de Lausanne (EPFL), Lausanne, CH-1015, Switzerland; 7 Synchrotron and Nanotechnology Department, Paul Scherrer Institute, Villigen, CH-5232, Switzerland

## Abstract

Optimisation problems typically involve finding the ground state (*i.e*. the minimum energy configuration) of a cost function with respect to many variables. If the variables are corrupted by noise then this maximises the likelihood that the solution is correct. The maximum entropy solution on the other hand takes the form of a Boltzmann distribution over the ground and excited states of the cost function to correct for noise. Here we use a programmable annealer for the information decoding problem which we simulate as a random Ising model in a field. We show experimentally that finite temperature maximum entropy decoding can give slightly better bit-error-rates than the maximum likelihood approach, confirming that useful information can be extracted from the excited states of the annealer. Furthermore we introduce a bit-by-bit analytical method which is agnostic to the specific application and use it to show that the annealer samples from a highly Boltzmann-like distribution. Machines of this kind are therefore candidates for use in a variety of machine learning applications which exploit maximum entropy inference, including language processing and image recognition.

**Maximum Entropy Decoding**. A universal problem in science and engineering and especially machine learning is to draw an objective conclusion from measurements which are incomplete and/or corrupted by noise. It has been long recognised^1^,^2^,^3^ that there are two generic approaches for doing this:

1. maximum a priori (MAP) estimation, which results in a unique conclusion which maximises the likelihood of being correct;
2. marginal posterior maximisation (MPM), which results in a probabilistic conclusion whose distribution maximises entropy.

The maximum entropy MPM approach is an implementation of Bayesian inference since prior knowledge about the expected distribution of the measurements is required. Maximum entropy modeling has been shown to be a highly effective tool for solving problems in diverse fields including computational linguistics^4^, unsupervised machine learning^5^, independent component analysis^6^, ecological modeling^7^, genetics^8^, astrophysics^9^, solid state materials physics^10^ and financial analytics^11^.

As an archetypal example of Bayesian inference we consider the decoding problem, and specifically the extent to which the maximum entropy MPM method outperforms the maximum likelihood MAP method when implemented on a programmable Josephson junction annealer. Decoding is a canonical, computationally hard optimisation problem. The goal is to extract information from a signal which has been corrupted by noise on a transmission channel. By adding *M* redundant bits to the *N* transmitted information bits it is possible to recover the data exactly provided that *M*/*N* exceed a noise-dependent threshold value set by Shannon’s theorem. Turbo codes and low-density parity check codes represent the current state-of-the-art for decoding and come close to reaching the Shannon limit. We choose the decoding problem since, as we show below, it maps directly onto a hardware implementation of the Ising spin glass and the prior knowledge required to maximise the entropy of the decoding information is precisely quantifiable and described by a single parameter.

**Ising Code**. Sourlas^12^ noted the algebraic similarity between parity check bits in information coding and magnetically-coupled spins in an Ising spin glass. He introduced the Ising code, in which *N* information bits 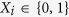 are mapped onto *N* spins 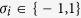. A set of 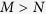 couplers ***J*** can be defined from these bits such that 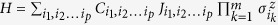 where 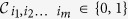 is the *connectivity* of an underlying graph and *m* is the locality of the graph. The set ***J*** of couplers is transmitted over a noisy channel. The maximum *likelihood* decoded word then corresponds to the ground state (*i.e*. zero temperature) spin configuration for the received (corrupted) set of couplers 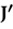. For 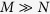 and large *m* the Ising code asymptotically approaches the Shannon limit.

The maximum *entropy* decoded word corresponds to the sign of the thermal average of the state of each spin at a finite temperature 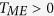 :


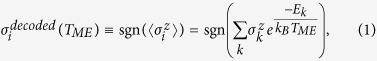


where 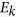 is the energy of a state and 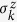 is its orientation (The usual normalisation term is omitted here since it is always positive.) Rujan^13^ showed that 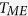 is given by the so-called Nishimori temperature^14^ which is a monotonic function of the magnitude of the channel noise and is therefore a noise dependent quantity. In other words, given prior information about the channel noise (and therefore about the likely distribution of the received set of couplers ***J′***), maximum entropy inference can be used to obtain better decoding performance than the maximum likelihood approach. More recently the role of quantum fluctuations on inferential problems including decoding have been considered^15^,^16^,^17^. The current paper does not seek to determine the role of quantum fluctuations, in finding an optimal solution; instead it considers the extent to which thermal fluctuations (which will necessarily be present in any real implementation of a Sourlas decoder) may be exploited.

**Programmable Annealer**. The D-Wave chip^18^,^19^ is a superconducting integrated circuit implementation of an Ising spin glass in a local (longitudinal) random field, 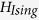, to which a local transverse field can be applied to relax the spins^20^. In the case where minimum energy (*i.e*. maximum likelihood) solutions are retained, its use in machine learning^21^,^22^ and a number of other applications^23^,^24^,^25^,^26^,^27^,^28^ has been proposed and/or demonstrated. There is significantly less work on exploiting excited states in computation. Their use was first analyzed in the context of characterizing graph structure^29^. Subsequently, the error correction protocol in^30^,^31^, used excited states to find the lowest energy state of an error-corrected Hamiltonian; this cannot be considered a maximum entropy application. It is also worth noting that^26^ used distance of found excited states from the ground state as a metric of success for the D-Wave annealer.

A time-dependent Hamiltonian describes the chip:


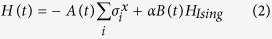


where


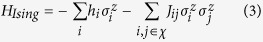


Here 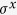 and 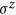 are the Pauli spin matrices and the 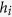 and 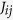 are user-programmable local fields and couplers respectively. *α* sets the overall Ising energy scale and is also user-programmable. The connectivity of the D-Wave machine is described by the so-called Chimera graph, 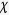, as shown in Fig. 1. The connectivity is sparse with all couplers being two-local. Each spin is coupled to at most either five or six other spins. The magnitude *A*(*t*) of the transverse field sets the scale of quantum fluctuations on the chip. During the course of a single optimisation run, *A*(*t*) is adiabatically reduced to near zero in a manner analogous to simulated annealing in which the scale of thermal fluctuations (*i.e*. the temperature) is reduced to zero. At 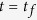 the system is described by 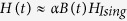 and the dynamics are fully classical. If the chip were operated at zero temperature then, in the absence of non-adiabatic transitions and control errors, at 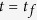 , the spins would be in the ground state configuration of 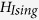. However, since the chip is operated at finite temperature 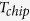, there is a non-zero probability that excited states will be occupied at 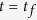. The non-zero occupation of the excited states suggests that the spin configuration which maximises the entropy of the system differs from the mean orientation in the ground state configurations.

The facts that the D-Wave chip operates at finite temperature and that the dynamics at the end of the annealing process are fully classical have led to a debate about the extent to which quantum mechanics plays any role in its computational output^26^,^27^,^32^,^33^,^34^,^35^,^36^,^37^,^38^,^39^,^40^,^41^,^42^. For the entropy to be maximised, however, it is only necessary that the final distribution of excited states follows the Boltzmann distribution, which is *by construction* the one which maximizes the entropy for a given total ensemble energy. In this context the long timescale of the annealing process can be seen as an advantage in that it allows the system to fully thermalise. Nevertheless the transverse field term in equation 2 may help prevent local minima from trapping the system as suggested in reference^42^,^43^.

In our experiments we perform *encoding* with conventional computational resources. The gauge symmetry of the Ising spin glass allows us without loss of generality only to consider a data word *X* consisting of a string of *N* ‘ones’. In the Ising code as described by Sourlas^12^ the transmitted codeword consists of the *M* couplers (here all ferromagnetic, 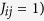. Based on Eq. 1 and 3 we note however that if 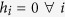 then for the noise free transmission channel, there are two trivial uniform solutions for the decoded value 
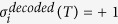
 or 
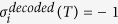
, due to the overall 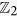 symmetry. To break this symmetry, in addition to transmitting the couplers over the noisy transmission line, we also transmit the values of the local fields 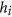, with assigned values 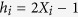. what we are effectively doing is sending both the N original information bits and the parity bits 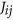 over the noisy channel. At the coding stage the coupling graph is selected to match the connectivity of the D-Wave chip for ease of subsequent decoding. The rate and distance of the Ising code as implemented on the Chimera graph depend upon the word length *N* (*i.e*. upon the number of spins) and are shown in Table 1.

We model transmission over the binary symmetric channel (BSC). Here the probability that any local field is received corrupted (*i.e*. 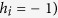 is equal to the probability that any coupler is received corrupted (*i.e*. anti-ferromagnetic, 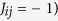 and given by the so-called crossover probability *p*. Corrupted bits and couplers are uncorrelated. For this channel the Nishimori temperature is


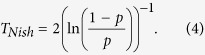


*Decoding* is performed by the D-Wave chip according to the annealing schedule which begins with 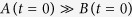 and ends with 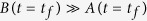 with 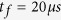. Here 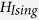 is defined by the bias fields 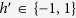 (corresponding to the received corrupted set of *N* data bits) and the couplers 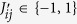 (corresponding to the received corrupted set of *M* couplers). Finite temperature decoding consists of repeating the annealing process many times to find the sign of the average of each spin:


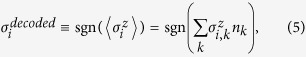


where 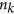 is the number of occasions that state *k* is occupied at the end of the anneal and 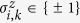 the orientation of spin *i* in state *k*. Under the assumption that the D-Wave machine samples from a Boltzmann distribution, one could in principle adjust its temperature 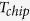 to maximise entropy. However since the machine is only calibrated at a fixed temperature (set by the base temperature of the dilution refrigerator), we instead vary the Nishimori temperature at the encoding stage and keep the machine temperature constant. Maximum likelihood decoding is the special case of equation 5 where only the spin configurations corresponding to the ground state (or multiple degenerate ground states) are considered.

For small systems we can compare the decoding performance of the D-Wave machine with an analytical model. In this case the spectrum of states corresponding to the received set of bias fields and couplers is calculated by summing exhaustively over all states; the population of these states is assumed to follow a Boltzmann distribution. We further define temperatures at which the value of 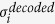 in Eq. 1 changes, which we call spin-sign transition temperatures. These transitions allow us to not only estimate the temperature which best fits the experimental data, but also to determine to what extent our data matches a Boltzmann distribution, which can be treated as a method of measuring equilibration.

**Connection to spin glasses**. It is worth asking questions about the Hamiltonians we use based on spin glass theory, which can be used as a way of gauging the ‘hardness’ of solving a typical instance. A spin glass phase which exists at finite temperature implies that finding the ground state is ‘hard’ using realistic (i.e. local) thermal fluctuations or software algorithms such as Monte Carlo and parallel tempering. It is important to note that this statement is in one sense stronger than the statement that a problem is NP-hard, because it is a statement about typical rather than worst-case hardness, but weaker in another sense because it only addresses one approach to solving the problem. It is worth noting additionally the recent propolsal for a general purpose algorithm which has been shown to be very efficient for solving typical instances from classes of Hamiltonians with zero temperature spin glass transitions, such as the chimera graph without fields^44^.

There have not been any studies of whether a Chimera graph in a field has a finite temperature spin glass phase. Zero field random Chimera graphs have been shown to not have a finite temperature spin glass transition^45^. There are many other examples however where a model has a finite temperature spin glass phase without a field, but the inclusion of a field causes this phase to vanish^46^,^47^. The 3D Ising Edward-Anderson model provides a concrete example of this phenomenon^48^,^49^. This, coupled with the numerical evidence that chimera graphs which are scaled up with fixed unit cell length will have the thermodynamic properties of a 2 dimensional graph in the large system limit^45^,^50^ suggest that a chimera graph in field will not have a finite temperature spin glass transition.

It is important to note that the set of Hamiltonians which we study here is *not* what is traditionally defined as a Random Field Ising Model (RFIM). RFIMs have the additional constraint that all 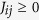. This subtle distinction is important, because it has been shown that RFIMs do not have a spin glass phase in equilibrium^51^,^52^. As can be done with any 2-body interaction Hamiltonian, our Hamiltonians can all be defined in a gauge where all of the local fields are in the same direction, and act as an effective uniform global field. They therefore can be considered a hybrid of a Mattis Spin glass^53^ and a “spin glass in a uniform field”, approaching the latter exactly when 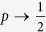. While there are open questions about the nature of the spin glass transition for a spin glass in a uniform field, it is generally accepted that (for some topologies) these models do still behave as spin glasses. In particular, it is an open question^54^ whether Replica Symmetry Breaking^55^ (RSB) provides an appropriate description of these models, or whether they are better described in a phenomenological “droplet” picture^56^,^57^. As for quantum annealing itself, the most relevant experiments have been performed in LiHoxY1-xF4, where the transverse field imposed in the laboratory introduces not only quantum fluctuations, but also internal random longitudinal fields^58^,^59^,^60^.

The techniques laid out in this manuscript provide a way of probing equilibration. If the spin-sign transition temperatures (where the value of Eq. 1 changes) which we calculate using exhaustive summing and tree decomposition methods could be calculated using a method which supports larger system sizes, and an interesting experimental or numerical system of such a size were available then our methods could prove a valuable tool to examine equilibration . It is also worth pointing out that, while we choose to examine spin-sign transitions due to the connection with the real world problem of decoding, an analogue of Eq. 1 for correlations, 
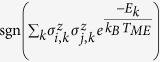
, and correlation-sign transitions could be defined in analogue to our spin-sign transitions. While spin-sign is only meaningful in models where the 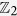 symmetry of the Ising model is broken, correlation-sign transitions carry no such restriction, and could be used with Hamiltonians with no field terms.

Even without having access to a method which can reliably calculate spin- or correlation- sign transitions for larger systems, generalizations of our methods could be interesting for example to examine whether equilibrium or non-equilibrium effects are the limiting factor in small instances of various benchmarking experiments, such as those performed in^61^.

## Experimental Methods

All decoding experiments were performed on the D-Wave Vesuvius processor located at the Information Sciences Institute of the University of Southern California. This processor contains 512 bits in an 8 × 8 array of unit cells. Due to fabrication errors, nine of the bits fall outside of the acceptable calibration range. Any unit cells containing one or more of these uncalibrated bits were therefore not used in our experiments. The annealing time 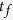 was fixed at 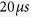. Each bit in each received corrupted word was mapped onto a randomly chosen gauge on the chip. To determine the bit-error-rate for a given value of crossover probability, we first sum the observed values of the orientation of each of the spins used and take the sign using the fact that Eq. 1 becomes 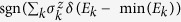 in the limit of 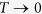. We then define the bit-error-rate as the probability with which the decoded spin orientation disagrees with the original message. Because we have chosen a trivial message, this amounts to calculating 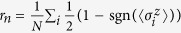 for each Hamiltonian 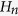 and performing a sum weighted by the probability 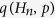 that this Hamiltonian will be generated. For a given crossover probability *p*, the bit-error-rate is given by


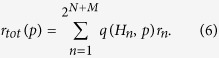


In practice, we simplify this calculation greatly by taking advantage of the fact that the value of 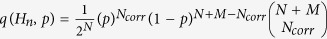, only depends on the number of couplers and fields which are corrupted, 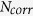, and the crossover probability *p*. We explain how we exploit this fact in Sec. 1 of the supplemental material.

### Macroscopic Analysis of Decoding

#### Single unit cell

**Figure 2:**
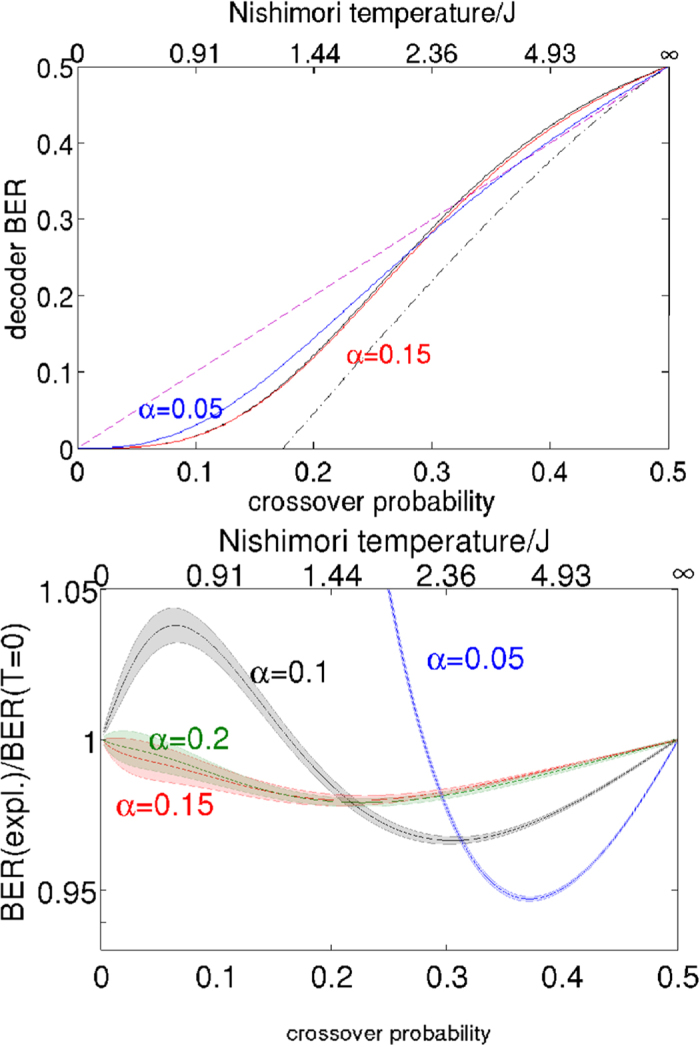
Top: Bit-error-rate (BER) for a single Chimera unit cells plotted as a function of channel crossover probability. The solid line black line represents theoretically calculated ground state decoding. Red line is experimental data with 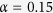 which corresponds to a coupling energy scale of 13.4 mK (at the point when the dynamics freeze, this energy scale was established based on freezing when the transverse field energy scale is 0.1 GHz as suggested in private communication with Mohammad Amin.) which is the order of the base temperature 17 mK of the cryostat. Blue line is experimental data with 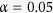 which corresponds to a coupling energy scale of 4.99 mK. The dot-dash line shows the Shannon-limited minimum achievable bit-error-rate for a decoder of rate 0.333, corresponding to the rate of the Ising code on a single unit-cell. The dashed line is a guide to the eye showing the locus of points where the decoder bit-error-rate is equal to the crossover probability. Bottom: Ratio of experimental decoding error rate to maximum likelihood decoding error rate. Shaded areas are one standard deviation estimated by bootstrapping. *α* = 0.1 corresponds to 8.9 mK and *α* = 0.2 corresponds to 17.8 mK.

The connectivity is defined by a single Chimera unit cell containing 8 spins. Hence the number of data bits is *N* = 8 and the number of couplers is *M* = 16. The decoded bit error rate is plotted as a function of the crossover probability, *p* (or equivalently the Nishimori temperature, 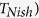 in the top subfigure of[Fig f1] Fig. 2. The decoder is useful (*i.e*. the decoded bit error rate is lower than *p*) for *p* < 0.327 but compares unfavourably with the Shannon-limited performance.

We now consider whether the maximum entropy approach is effective in reducing the bit error rate. The bottom subgraph of Fig. 2 shows the ratio of the experimental to theoretical bit-error-rates for zero temperature.We mimic the effect of changing the decoding temperature by changing the energy scale *α* of the Ising Hamiltonian in Eq. 2; here higher values of *α* correspond to lower decoding temperatures. A similar technique has been applied in^32^ in the context of identifying quantum effects. At *α* = 0.05 and *α* = 0.1 the D-Wave finite temperature decoding compares poorly with ground state decoding at low noise levels but is superior at higher values of the crossover probability. As the effective decoding temperature is lowered (*e.g. α* = 0.2) we reach the regime where finite temperature decoding always outperforms maximum likelihood decoding. Further reducing the decoding temperature has no effect on the bit-error-rate for reasons which we will explain later.

It is worth noting that the maximum entropy method, which relies simply on measuring thermal expectation values, never outperforms the maximum likelihood “ground state” calculations by more than 10%. This most likely is a reflection of the extent to which thermal fluctuations for a quadratic Ising model can mimic the effects of higher order spin couplings (i.e. beyond quadratic) which optimized Sourlas codes for error correction contain.

**Figure 3:**
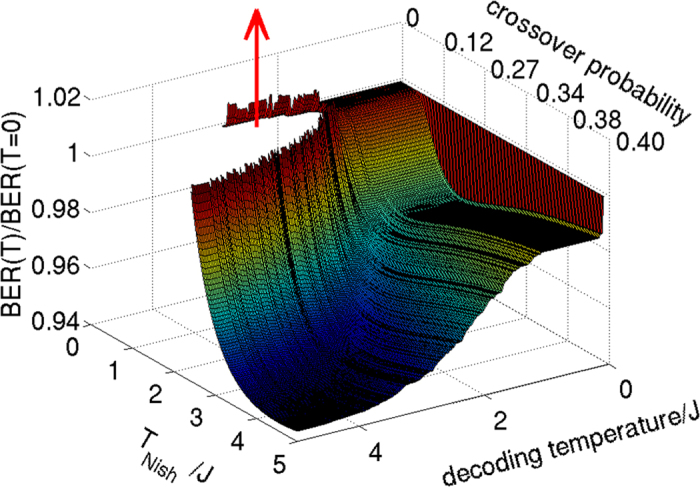
Analytically calculated bit-error-rate (BER) for a single unit-cell of the Chimera graph, plotted as a function of the decoding temperature and the Nishimori temperature. The bit-error-rates are normalised with respect to those obtained using maximum likelihood decoding.

We now compare the finite temperature experimental result of Fig. 2 with the analytical result obtained by exhaustive sums assuming a Boltzmann distribution. At low Nishimori temperature (*i.e*. low channel noise) the Bayesian approach fares badly by comparison with the maximum likelihood approach. In this regime the correct error-free codeword is typically the closest allowed codeword (in the Hamming sense) to the received corrupted codeword, and the error-free decoding corresponds to the ground state spin configuration. As the channel noise increases, however, the maximum entropy approach outperforms ground state decoding for the quadratic Hamiltonian used here. It can be seen therefore that, at least qualitatively, the D-Wave machine behaves as a maximum entropy decoder. It has been proven^14^,^62^ that the bit error rate is minimised when the decoding temperature is equal to the Nishimori temperature 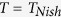. Note however that owing to the fact that this curve is discontinuous as a function of *T*, the converse is not necessarily true – *i.e*. for a fixed value of *T* it is *not* necessarily true that the optimum will be at 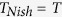. See Sec. 2 of the supplemental material accompanying this document for a graphical demonstration that the results from reference^14^,^62^ do in fact hold for the data in Fig. 3.

The discontinuity of the bit error rate as a function of decoding temperature results from spin-sign transitions as described earlier. Of particular note is the plateau at normalised temperatures below 1 unit, suggesting that there are no such spin-sign transitions in this temperature range. It is for this reason that the experimental data do not differ significantly from each other for 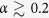. An analysis based on these transitions follows later in the manusript. Simulations (not shown, but see Sec. 3 of the supplemental material) indicate that the magnitude of the discontinuities and the temperature width of the plateau both reduce as the system size increases. The experimental data in Fig. 2 reproduce the analytical data rather well, confirming that the population of the excited states at the end of the annealing process is at least reasonably well described by a Boltzmann distribution.

**Figure 4:**
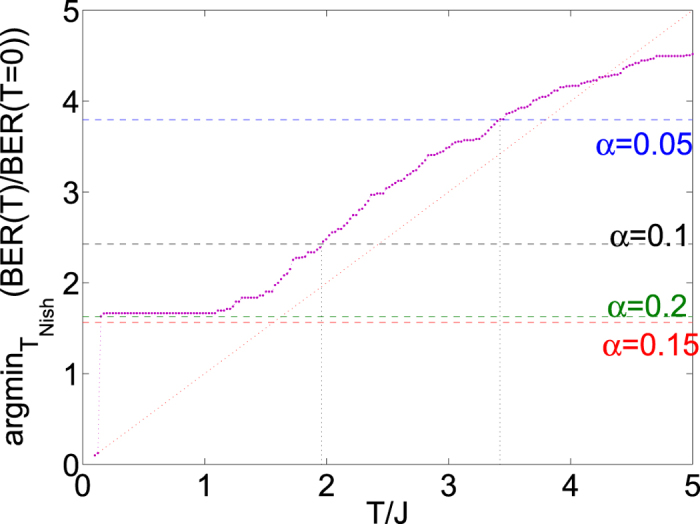
The analytically calculated value of the Nishimori temperature at which the bit-error-rate (BER) ratio is minimised (i.e. entropy is maximised), plotted as a function of the decoding temperature for a single Chimera unit cell. The dotted line is included as a guide to the eye. Note that the data points at 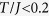 are a numerical artifact caused by finite machine precision. The horizontal dashed lines show temperatures obtained by numerically extracting the minima from the experimental results from the annealing device in Fig. 2. As explained in Sec. S1, these curves are given by a polynomial, the minimum can therefore be found using a standard algorithm.

We further perform a direct comparison between the theoretical data in Fig. 3 and the experimental data in Fig. 2. Specifically, we consider the value of 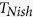 at which the ratio of the maximum entropy to maximum likelihood error rates is the smallest. Figure 4 compares the experimentally observed minima to the theoretical predictions. For *α* = 0.2 and *α* = 0.15 the predicted minima are within a large ‘plateau’ and therefore the location of the minimum is well explained by a wide range of temperatures. For *α* = 0.1 and *α* = 0.5 however we can make such an estimate, which puts the “effective temperature” for *α* = 0.1 at around 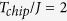 and the value for *α* = 0.5 at around 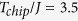. We later perform analysis which allows us to make a much more accurate estimate of these temperatures.

### 4 × 4 Array of Unit Cells

**Figure 5:**
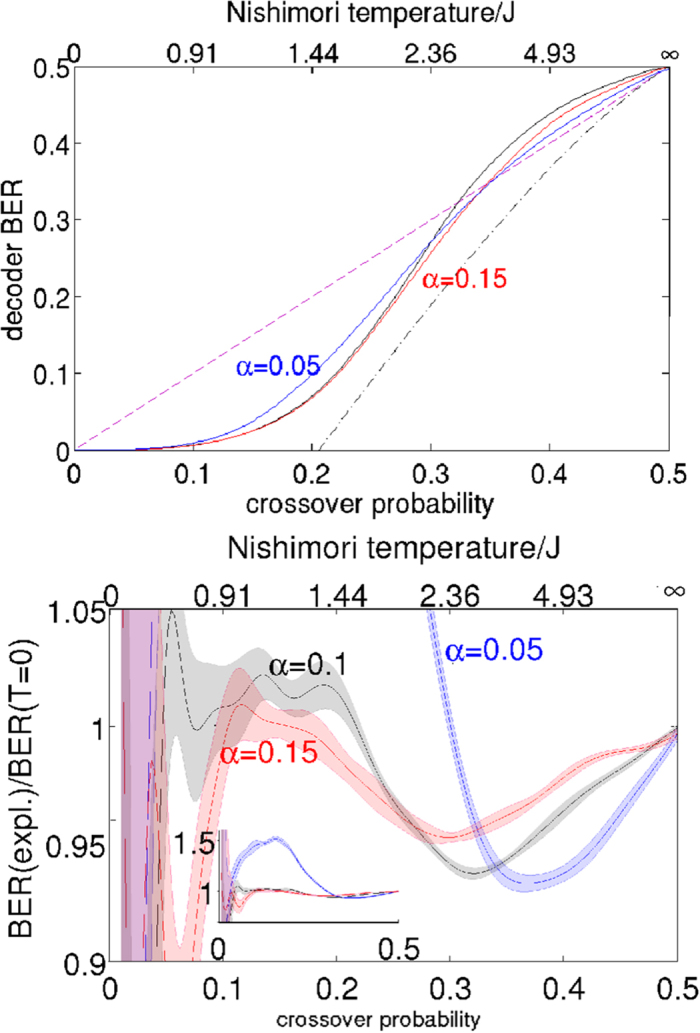
Top: Bit-error-rate (BER) for a 4 × 4 array of Chimera unit cells plotted as a function of channel crossover probability. The solid line black line represents theoretically calculated ground state decoding for the 4 × 4 array. Red line represents experimental data with *α* = 0.15 which corresponds to a coupling energy scale of 13.4 mK (at the point when the dynamics freeze) which is the order of the base temperature 17 mK of the cryostat. Blue line represents experimental data with *α* = 0.05 which corresponds to a coupling energy scale of 4.99 mK. The dot-dash line shows the Shannon-limited minimum achievable bit-error-rate for a decoder of rate 0.276, corresponding to the rate of the Ising code on a 4 × 4 chimera graph. The dashed line is a guide to the eye showing the locus of points where the decoder bit-error-rate is equal to the crossover probability. Bottom: Ratio of experimental decoding rate to maximum likelihood decoding rate. Shaded areas are one standard deviation estimated by bootstrapping. *α* = 0.1 corresponds to 8.9 mK.

To demonstrate that the maximum entropy approach is effective for larger systems, let us now consider decoding for a 4 × 4 array of Chimera unit cells. These data, with 128 information bits and 352 couplers using the D-Wave chip are shown in Fig. 5. In this example the qualitative features of the analytical result for an 8-bit word are reproduced. For each value of *α* there is a wide range of crossover probabilities where the experimental system outperforms exact zero temperature calculations. The feature where a low *α* system performs very poorly compared to the zero temperature result is also qualitatively reproduced.

It is worth noting that the difference between the maximum entropy approach and the maximum likelihood approach is significantly more dramatic for *α* = 0.15 in this case than it was for the single unit cell. This may partially be due to the fact that the rate of this code is lower, but is also probably related to the fact that the larger system with more complex topology allows for a greater richness of possible thermal fluctuations.

### Microscopic Analysis

We have established that the macroscopic behavior of spin decoding on our experimental system is quite similar to what would be expected for a thermal distribution. While the macroscopic investigation demonstrates that the chip can be used for finite temperature decoding, it does not provide strong evidence on how suitable the chip may be for other maximum entropy tasks. To answer this question we need to examine whether or not the individual spin orientations on the annealing machine look similar to those expected from a Boltzmann distribution.

The Boltzmann distribution is uniquely and by construction that which maximizes entropy for a given energy cost. In the case of maximum entropy tasks we can think of this as optimizing robustness to uncertainty, given that we are willing to sacrifice a given amount of optimality in our solution. The closer our result is to Boltzmann, the closer the result is to optimizing this tradeoff. In the next section we introduce the theory behind our microscopic analysis, and apply it to our experimental data in the section following that.

It is important to note that, unlike the previous section, the microscopic analysis is independent of the intended application. We have chosen a decoding example for this study because the mapping onto the D-Wave chip is trivial. The results of this section however apply equally well for other, technologically useful maximum entropy applications.

### Microscopic theory of Spin-Sign transitions

To examine the performance for maximum entropy tasks on a microscopic scale, we must first have a theoretical understanding of what is expected for a Boltzmann distribution. For a system which consists of a finite number of spins the value of 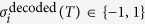 can only change a finite number of times, 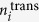, on the interval 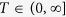. For this reason we can uniquely characterize the decoding of each spin by the set of temperatures 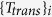 at which the value of 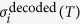 changes, and the value of 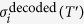 at any single temperature 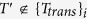. We will refer to each of these temperature dependent changes in orientation as a *spin-sign transition*. It may often be the case that for a given Hamiltonian a spin has no spin-sign transitions and therefore 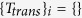. For these spins 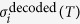 is independent of *T* and maximum entropy decoding can be considered trivial. There can further be Hamiltonians in which this is the case for all spins, for example this must be true for any Hamiltonian in which all couplers and fields can be satisfied simultaneously.

To analyze the performance for a maximum entropy application, we choose to focus on the non-trivial cases of spins which have at least one spin-sign transition. Let us define the low temperature decoding of a spin as 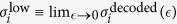. Note that we define this as a limit to avoid ambiguity in cases where 
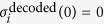
. For an experimental system we can define the probability of agreeing with the low temperature result over many repetitions of the experiment 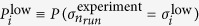 where 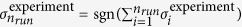 and 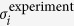 is the result of a single experimental run. The probability of agreeing with the low temperature result, 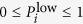 takes a continuum of values rather than just 0 or 1 because individual experiments do not always yield the same results. We define 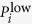 in this way for two reasons. First, the user interface to the device places an upper limit on 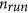, so we cannot be confident that the result of a single experiment is statistically significant. Second and more importantly, each experiment is subject to a set of control errors, which do not change significantly during the experiment, we therefore must average over multiple experiments in different gauges to get the true sampling behavior. Because of these control errors, it is not necessarily true that 
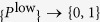
 even in the limit 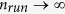.

In general, a single spin can undergo many spin-sign transitions. If we assume that a sufficient number of experiments have been performed to be statistically confident in the experimental result, 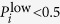 implies that 
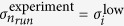
. This does not generally demonstrate that the experimental system is at a low temperature, only that it has undergone an even number of spin-sign transitions from 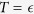. Conversely 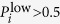 implies an odd number of transitions.

Let us now consider how to apply the methods to real experimental data. If we are at a low enough temperature that we can neglect the possibility that a spin has multiple spin-sign transitions below that temperature, and assume that 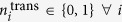, then experimental measurements of 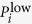 allow for a convenient way to check if the lowest temperature decoding transition has occurred. If 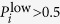 than we can conclude that the transition has happened, otherwise we can conclude that it has not. Now consider experimentally measuring 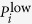 for each bit in a series of randomly generated Ising Hamiltonians, and plotting the result as a function of the theoretically calculated spin-sign transition temperature, 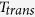. If the experimental system produced a perfect Boltzmann distribution at a temperature 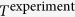, then in the limit this plot would look like a step function going from one to zero centered at 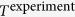. Reducing 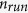 to a finite value will broaden out this sharp transition, but will not affect the temperature at which the plot crosses 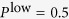. We can calculate the temperature dependence of the spin orientations by explicitly constructing the Boltzmann distribution. We can then apply the central limit theorem to calculate the theoretically expected 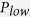:


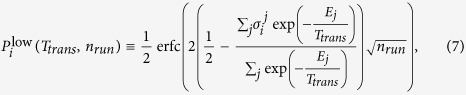


where 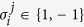 is the orientation of bit *i* within state *j*.

Let us now consider the transitions for all single cell Hamiltonians with 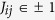 at some energy scale *α*. At first sight the calculation of all of these spin-sign transitions temperatures seems like a monumental task given that naively there should be 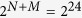 different Hamiltonians. However, only Hamiltonians which cannot be mapped into each other by gauge or symmetry transformations can have different spin-sign transitions. The gauges allow us to reduce the number of Hamiltonians we examine to 2^16^ while the symmetries of the unit cell allow us to further reduce this number to 192. We are therefore able to experimentally test each of these Hamiltonians on the D-Wave chip, as described in the next section. It is important that, in the case of the single unit cell, there are no spins which show more than one spin-sign transition at finite temperature. The transition plot should therefore be a valid way to analyze the decoding at any temperature without having to consider errors due to multiple transitions.

The next natural question concerns the performance on larger systems. For this purpose, we consider a 4 × 4 chimera graph with 128 qubits. While the calculation of the spin-sign transition temperatures cannot be performed by exhaustive search the way the single unit cell was, it can be efficiently solved using a bucket tree elimination (BTE)^63^. A software sampler based on BTE allows us to estimate the orientation of each spin as a function of temperature. From this estimate we can extract 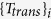 and 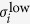 for each bit. For a detailed explanation of our methods see Sec. 4 of the supplemental material. For spins with a ground state orientation of zero, the orientation curve as a function of *T* remains very close to zero at low temperatures. This is problematic for our analysis because statistical error can cause us to detect a large number of spurious transitions. For this reason we exclude these data from our analysis.

We quantify the performance of the chip for maximum entropy tasks on a larger system in two ways. First we can compare all Hamiltonians with a Boltzmann distribution at the same temperature, and define


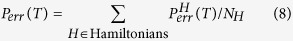


where 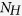 is the number of Hamiltonians examined and


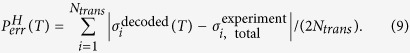


Second we admit the possibilities that different Hamiltonians freeze at different times and examine 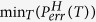 for each Hamiltonian individually. It is important to emphasize again that we only include bits with at least one spin-sign transition in this analysis to avoid the data being overwhelmed by spins which behave trivially.

### Microscopic results

**Figure 6:**
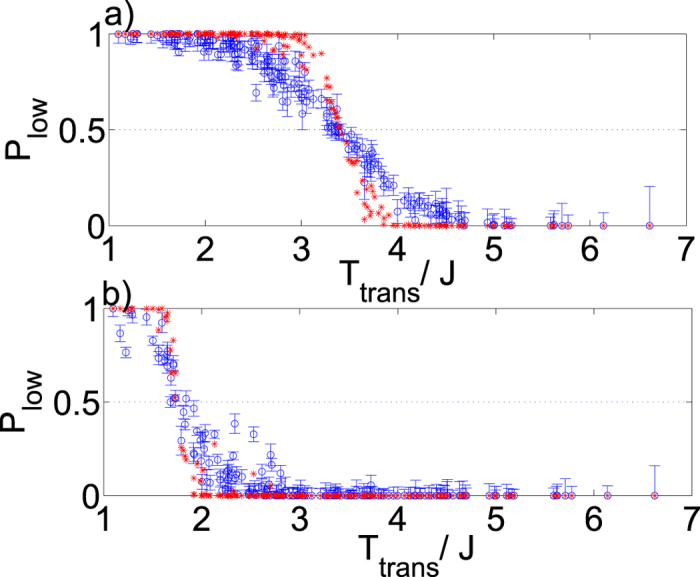
Single unit cell decoding transitions. Circles with errorbars (blue) are experimentally-measured values of the probability of each bit decoding to the predicted low-temperature result plotted as a function of the calculated spin-sign transition temperature. Error bars show one standard deviation. Asterisks (red) are the calculated behavior for a Boltzmann distribution with a temperature determined by fitting to Eq. 7. Dotted line at 0.5 is a guide for the eye. (**a**) *α* = 0.05; in this case the fitted temperature is 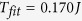. (**b**) *α* = 0.1; in this case the fitted temperature is 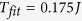.

In Fig. 6 we show the experimentally-measured probability that each bit in a single Chimera unit cell decodes to the calculated low temperature orientation, plotted as a function of the calculated spin-sign transition temperature. We also show the theoretical dependence obtained by assuming that the annealing device precisely follows a Boltzmann distribution - i.e. equation 7. The data plotted in Fig. 6 demonstrate that for this example the device should perform maximum entropy tasks quite well. However the data differ from what would be expected from a device which samples a pure Boltzmann distribution: in both plots the transition is significantly broader than the pure Boltzmann result. This effect can most likely be attributed to control errors, which cause random deviations in the couplers and the fields. It is also worth noting that especially in 6(b) there are some individual transitions which decode correctly but deviate strongly from what we would expect from a Boltzmann sampler. These could be due to dynamical effects similar to the ones explored in^64^. For this reason the Hamiltonians associated with these transitions may warrant future study. In the interest of future work, we include these Hamiltonians in Sec. 5 of the Supplemental Material.

The fits performed for the single unit cell can provide us an estimate of a phenomenological temperature for the device which seems quite robust. We can use this effective temperature combined with the known annealing schedule and physical temperature of the device to find the parameters at the ‘freeze time’ when the dynamics effectively stop. Table 2 displays these results. As one may intuitively expect, the system appears to freeze earlier for larger values of *α*. Although the difference in apparent temperature is relatively small, it corresponds to freezing with a much higher value of transverse field. We note however that in all cases the transverse field at the freeze time is much weaker than the couplers.

**Figure 7:**
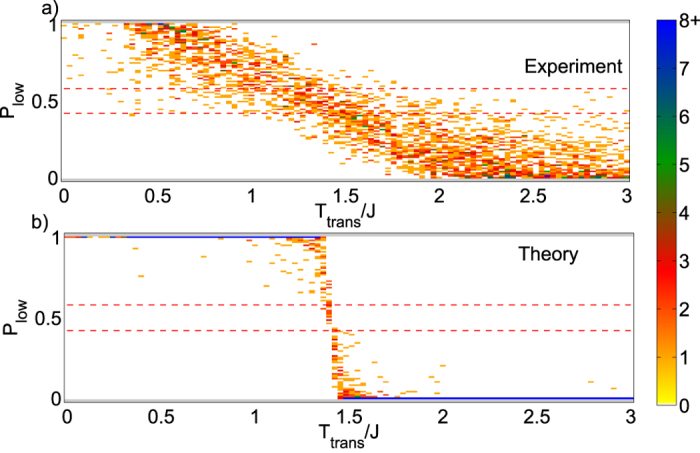
Densities shown in (**a**) are counts for experimentally-measured probabilities of decoding to low temperature result on the y-axis and theoretically determined transition temperatures on the x-axis. (**b**) is the same, but where the values on the y-axis are determined theoretically using Eq. 7 and 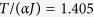. Both plots are for a 4 × 4 chimera with *α* = 0.15. Data are based on 145 randomly chosen Hamiltonians with 200 flipped bonds or fields. For each Hamiltonian 100 sets of 1000 annealing cycles are performed. 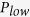 is the fraction of these sets of annealing cycles for which the spin agrees with the low temperature result. Dashed (red) lines are the distance from 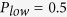 where data become statistically significant at the 95% level. Note that this density plot is only for spins with a single 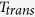; spins with multiple transitions have been observed but are excluded from these figures.

**Figure 8:**
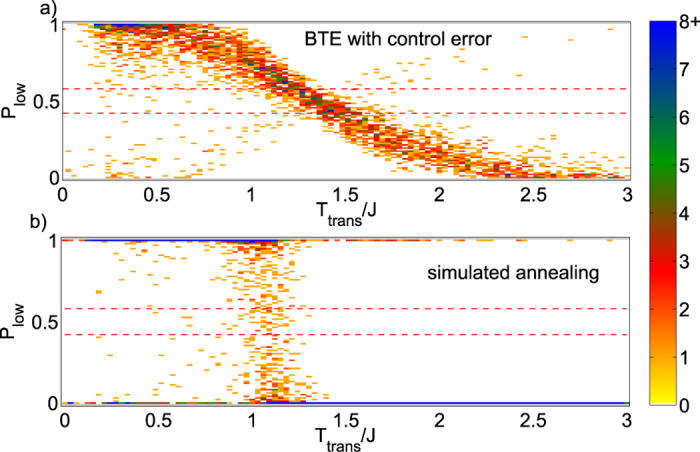
Top: bucket tree elimination sampled over independent control error of 5% J in the fields and 3% J in the couplers. These data represent perfect equilibration subject to control error. Bottom: Simulated Annealing (SA) with 10,000 total updates and no control error. These data were taken with a linear sweep starting from 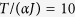. The error in these data come from failure to equilibrate. These data were calculated based on 1000 samples per run as were used experimentally. Dashed (red) lines are the distance from 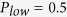 where data become statistically significant at the 95% level.

Unlike the case of the single unit cell, we cannot possibly exhaustively sum over all inequivalent Hamiltonians for a 4 × 4 unit cell block. We can however randomly sample Hamiltonians and look at the transitions. Figure 7(a) displays such a plot. As before we can compare with what would be expected for a machine which samples an ideal Boltzmann distribution (with the same number of samples per run) shown in, Fig. 7(b). From comparing 7(a,b) we observe that the step function shape is broadened much more than expected from finite sample size alone. The broadening is likely due to control errors (i.e. errors in the specification of 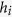 and 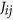 on the chip), which are different in each run and therefore would be expected to create broadening even if the number of samples per run is large. This is confirmed by simulations in which random control errors are added to the fields and couplers as shown in Fig. 8(a).

In Fig. 8(b) we show the results of simulated annealing in the absence of any control errors. By limiting the simulation to a total of 10,000 updates we see that failure to equilibrate produces a signature in which spins consistently decode with an orientation different from the one expected from Boltzmann statistics at the temperature which the system is operated. We do not observe this signature experimentally, which suggests that the device is in fact reaching equilibrium, which is consistent with the conclusions of reference^45^. Our experimental data do however display a clear signature of control error, which manifests itself in a broadening of the transition between low and high temperature decoding. As Fig. 8(a) demonstrates, this signature can be reproduced using BTE to calculate perfect equilibration in the presence of realistic control error. For further discussion comparing BTE and SA results, see Sec. 6 of the supplemental material accompanying this paper. The astute reader will note that Fig. 8(b) demonstrates a transition which is apparently shifted to a lower temperture. We suspect that this is due to the character of the local minima in which this simulation becomes trapped. It would be interesting to investigate this feature further, but such an investigation is beyond the scope of this work.

We now want to compare the data used to create Fig. 7 directly with those expected for an ideal Boltzmann distribution. For a given temperature and a given Hamiltonian we can calculate the expected decoding with the Boltzmann distribution and divide each bit with a transition into two categories, the ones which should decode to 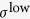 and those which should not, or equivalently those which have undergone even and odd numbers of transitions (including 0) from 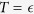. Only some of the experimental data are statistically significant. We consider only which are far enough away from 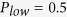.

**Figure 9:**
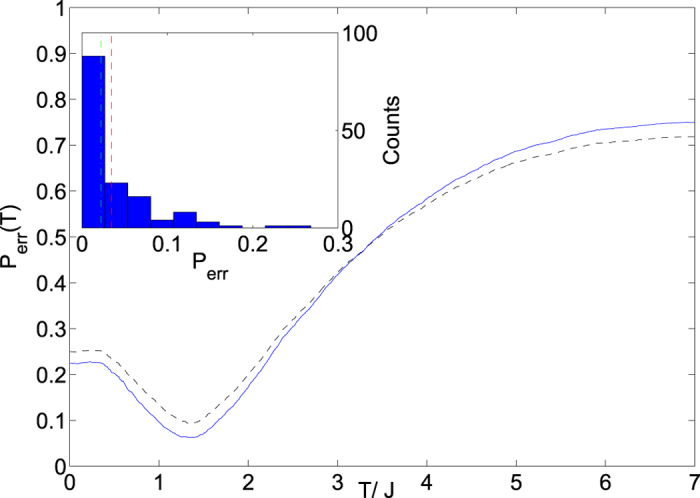
The main plot depicts the probability that the experiment finds an erroneous result for decoding a given spin assuming the underlying values of T/J given on the x-axis. Note that this is the same data set as Fig. 7(a) but including spins with multiple transitions. Rate of decoding errors versus temperature, dashed (black) line is all data, while solid (blue) line represents only the data which are statistically significant at the 95% level. Inset: Histogram of error rates at the best temperature for each Hamiltonian, 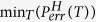. Dot-dashed lines are median, dashed lines are mean. A summary of relevant quantities extracted from these data can be found in Table 3.

We plot 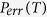 in Fig. 9. It shows a temperature for optimum performance which roughly agrees with our analysis of the single unit cell. A simple way to analyze the performance is to find the single temperature where the total performance summed over all of the Hamiltonians is the best. An alternate approach is to allow for the fact that different Hamiltonians may become ‘frozen’ at different points near the end of the annealing process and therefore different temperatures. Under this approach, 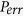 should be minimized for each Hamiltonian individually. A histogram of these data appears in the inset. From this histogram we notice that the device appears to perform quite well typically, but occasionally performs rather poorly.

We list relevant performance metrics for different values of *α* in Table 3. The table shows better performance by all metrics for larger values of *α*, again consistent with control errors being the limiting factor since at smaller *α*, the control errors will be more significant compared to the energy scale of the Hamiltonian. On the other hand, if the performance were limited because the dynamics were unable to reach equilibrium than we would expect to see the opposite trend, as smaller energy barriers would mean that the system is more able to reach equilibrium.

## Conclusion

We have shown that maximum entropy approaches to decoding, implemented using the D-Wave chip programmable Josephson junction annealer, can result in somewhat improved accuracy. This confirms that useful information can be extracted from the excited states (which, in any real machine at finite temperature, will have non-zero occupation) at the end of the annealing process. Our results are applicable to a wide range of problems in machine learning and optimization provided knowledge of prior information. Applications for which the maximum likelihood method is known to perform poorly or those where marginal benefits are valuable would be most suited to the current D-Wave architecture which features two-local couplers. An extension of the architecture to allow *m*-local couplers (with *m* ≥3) would allow the Shannon limit to be approached in decoding applications reference^65^,^66^.

We further show using analysis on the individual spin level that the experimental device produces a distribution which is Boltzmann like and therefore suitable for maximum entropy tasks. Additionally we demonstrate that the most probable limiting factors for such tasks are control errors, rather than equilibration dynamics. While our work has focused on the exploitation of the D-wave processor as a classical thermal annealing device, more experiments (e.g. by varying the annealing schedule) to determine the role (if any) of quantum fluctuations on maximum entropy inference using the D-Wave processor would be illuminating. We also note that generalizations of the methods we have outlined here may be useful to study disordered magnets such as spin glasses.

## Additional Information

**How to cite this article**: Chancellor, N. *et al*. Maximum-Entropy Inference with a Programmable Annealer. *Sci. Rep*. **6**, 22318; doi: 10.1038/srep22318 (2016).

## Supplementary Material

Supplementary Information

## Figures and Tables

**Figure 1 f1:**
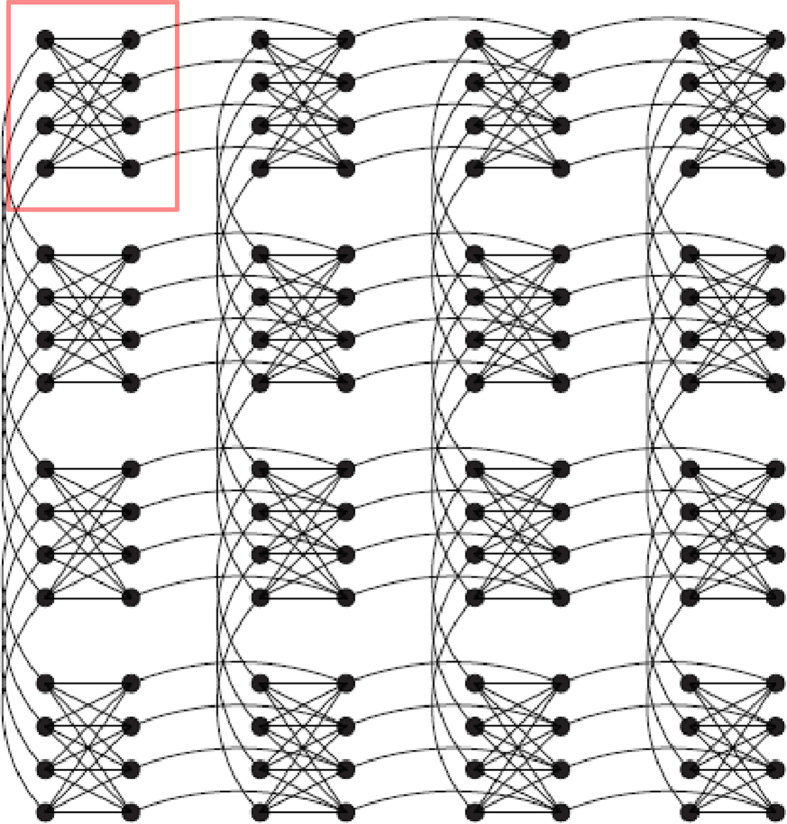
Chimera graphs used for this study. The full figure shows a 4 × 4 array of unit cells each containing eight spins, a single example of which is shown in the rectangle at the top left of the figure. Each dot represents a spin and each line a coupler.

**Table 1:** Table of relevant quantities for different mappings.

Word length, *N*	Chimera mapping	No. of couplers, *M*	Rate of code *N*/(*M* + *N*)	Distance of code
8	Single unit cell	16	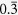	5
128	4 × 4 array of unit cells	352	0.276	6

**Table 2:** Table of effective temperatures and corresponding parameters at the freeze time.

*α*	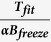	*B*_*freeze*_ GHz (mK)	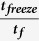	*A*_*freeze*_ GHz (mK)
0.05	0.170	2.08 (99.8)	0.748	0.0453 (2.17)
0.1	0.175	2.02 (96.9)	0.733	0.0530 (2.54)
0.15	0.180	1.86 (89.3)	0.696	0.0844 (4.05)

By fitting experimental data to Eq. 7 we can extract the ratio of the temperature to the strength of the couplings 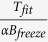. Because the temperature is fixed during the annealing process and the coupling changes monotonically with a known annealing schedule we are able to extract the value of the Ising energy scale 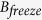 at which the system can no longer equilibrate. We then use the known annealing schedule to extract the freeze time, 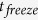, and the transverse field energy scale at the freeze time, 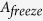. The device base temperature is 17 mK.

**Table 3:** Different metrics for the error rates at different values of *α*.

	Same T		Diff. T 95% sig.	
*α*	overall	95% sig.	mean	median
0.05	14.69%	9.30%	5.78%	3.03%
0.1	11.02%	8.15%	5.07%	2.82%
0.15	9.30%	6.23%	3.48%	2.22%

Same T indicates minimisation with the constraint that all Hamiltonians be modeled with the same effective temperature, while diff. T indicates that each one is allowed a different temperature. The data for ‘same T’ with *α* = 0.15 are the minima of the two curves in Fig. 9 (main figure) while the ‘diff. T’ data are the mean and median illustrated as the red and green line respectively in the inset of the same figure.
