# Supplementary material for: Maximum-Entropy Inference with a Programmable Annealer
Source: Sci Rep. 2016 Mar 3;6:22318. doi: 10.1038/srep22318 (PMC4776239; doi:10.1038/srep22318)
Supplement: Supplementary Information [file srep22318-s1.pdf]

Supplemental Material for: Maximum-Entropy Inference with a Programmable Annealer

Nicholas Chancellor*", Szilard Szoke^, Walter Vinci'†, ^ Gabriel Aeppli‡ and Paul A. Warburton"^

December 24, 2015

"London Centre For Nanotechnology 19 Gordon St, London UK WC1H 0AH
^Department of Electronic and Electrical Engineering, UCL, Torrington Place London UK WC1E 7JE
' University of Southern California Department of Electrical Engineering 825 Bloom Walk Los Angeles CA. USA 90089
†University of Southern California Center for Quantum Information Science Technology 825 Bloom Walk Los Angeles CA. USA 90089
‡Department of Physics, ETH Zürich, CH-8093 Zürich, Switzerland
Department of Physics, École Polytechnique Fédérale de Lausanne (EPFL), CH-1015 Lausanne, Switzerland
Synchrotron and Nanotechnology Department, Paul Scherrer Institute, CH-5232, Villigen, Switzerland

1 Calculation of Continuous decoded bit-error-rate Curves

Naively, it would appear that to plot message bit-error-rate versus crossover probability curves we would need to take a statistically significant sample for each value of T_{Nish}. However in this section we demonstrate that this is in fact not the case, by a careful choice of how we collect and analyze the experimental data, we can efficiently calculate the final bit-error-rate for *any* value of T_{Nish}. This method allows us to plot continuous curves, as displayed in Fig. 4 of the main text.

Let us first consider the general form of the Hamiltonian for our Ising system $H = \alpha(-\sum_i h_i \sigma_i^z - \sum_{i,j\in\chi} J_{ij}\sigma_i^z\sigma_j^z)$, subject to the constraint that $h_i \in \{-1,1\}$and $J_{ij} \in \{-1,1\}$. Due to this constrain there are a finite number of Hamiltonians which can be generated by the noisy channel, let us call this set $\{H\}$. For each $H_n \in \{H\}$we can count the number of corrupted bits in the recieved codeword $N_{corr}^{(n)} \equiv \sum_i \delta_{h_i^{(n)},-1} + \sum_{i,j\in\chi} \delta_{J_{ij}^{(n)},-1}$. Because all bits are

subject to the same crossover probability, p, the total probability of having a given number of bits flipped can be written as

$$q(H_n, p) \equiv q(N_{corr}, p) = \frac{1}{2^{N+M}}(p)^{N_{corr}}(1-p)^{N+M-N_{corr}}\binom{N+M}{N_{corr}}, \quad (1)$$

where $N + M$ is the total number of elements (couplers and fields) in the Hamiltonian. We can assign to each Hamiltonian a bit error rate r_n, for the purposes of this discussion $\{r\}$ could have either been calculated (for example by exhaustive summing or BTE) or obtained experimentally. We now write the total bit-error-rate as a function of crossover probability

$$r_{tot}(p) = \sum_{n=1}^{2^{N+M}} q(N^{(n)}_{corr}, p)\, r_n. \quad (2)$$

We now observe that$N_{corr} \in \{0, 1, 2 \ldots N+M\}$ so for a given value of p there are only $N+M+1$ possible values of $p'(N_{corr}, p)$. Based on this observation we can group these terms together to write down the total decoded bit-error-rate

$$r_{tot}(p) = \sum_{s=0}^{N} q(s,p)\binom{N+M}{s}^{-1} \sum_{n'=1}^{\binom{N+M}{s}} r_{s,n'} = \sum_{s=0}^{N} q(s,p)\,\bar{r}_s, \quad (3)$$

where $r_{s,n'}$is the list of bit-error-rates for all Hamiltonians with $N_{corr} = s$ and N'_s is the number of Hamiltonians sampled in sector s. Based on the formula given in Eq. 3we can make several observations. Firstly we not that $\bar{r}_s$ does not depend on p, and q does not depend on any of the decoding rates, therefore knowing the $N + M + 1$ values of$\{\bar{r}\}$ allows us to easily calculate $r_{tot}(p)$ for any value of p. Secondly, we note that $\bar{r}_s$ need not be an exhaustive sum over all possible Hamiltonians with s corrupted elements; a good approximation of $r_{tot}(p)$ could be obtained using $\bar{r}_s$extracted from a representative sample.

2 Confirmation that $T_{Nish} = T$ is the optimal decoding temperature for our theoretical data

It is not immediately obvious that Fig. 3 obeys the famous result by Nishimori that optimal decoding happens at $T_{Nish} = T$. For this reason we have plotted several slices of that figure in the T direction along with the value along the line where $T_{Nish} = T$ in Fig. S1. We immediately note that in that plot the curve representing $T_{Nish} = T$ crosses the other curves at there gloabal minima, thereby confirming that our theoretical data do in fact agree with this result. It is worth noting however that the converse of this famous result is not necessarily true, the minimum in the T_{Nish} direction does not necessarily occur at $T_{Nish} = T$.This arises from the fact that the decoding curves are discontinuous in the T direction.

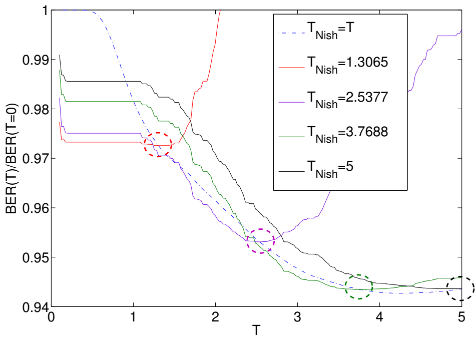

Figure S1: Theoretically predicted ratio of finite temperature bit-error-rate to zero temperature bit-error-rate versus T for a variety of Nishimori temperatures. Crossings at minimum values are circled for clarity. Here BER is shorthand for bit-error-rate.

3 Effect of system size on decoding

As we discussed in the main text, the plateau which can be seen in Figs. 3 and 5 of the main text shinks and the discontinuities in the ratio plotted in Fig. 3 decrease as the system size grows. The former can be seen by examining the number of expected spin-sign transitions for the 4x4 chimera versus the temperature at which the transition occurs. As Fig. S2 demonstrates, there are clearly spin-sign transitions within the 'plateau' region which was present for the single unit cell. It is worth noting that transitions in this region are also visible in Fig. S4.

To demonstrate the decrease in the size of the discontinuities with system size, we produce the equivalent of Fig. 3 in the main text but for a truncated version of the Chimera unit cell in which a bit has been removed from each half of the bipartite graph. As Fig. S3 demonstrates, the discontinuities become much more severe.

4 Estimation of spin-sign transitions for 4x4 Chimera

To estimate the temperatures at which spin-sign transitions occur, we use Bucket Tree Elimination (BTE) code which has been provided by D-Wave Systems Inc. This code acts as a fair Boltzmann sampler and can be made to provide an unbiased sample of N_{samp} spin states at a given temperature T. We use $N_{samp} = 10^5$. We then sample 200 temperatures between $T = 0$ and $T = 7$ to determine spin orientations. Fig. S4 gives an example of such data.

For each curve in Fig. S4 we can calculate the spin-sign transitions by first taking a running average over 5 neighboring points to suppress multiple spurious transitions and then using linear interpolation to find the zero crossings. An example of this procedure is demonstrated in Fig. S5. This method however

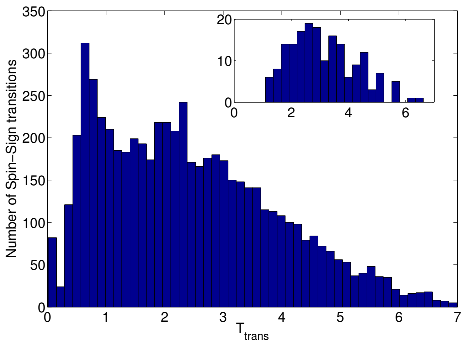

Figure S2 : Histogram of the number of spin-sign transitions versus transition temperature for 145 Hamiltonians with 200 corrupted bits each. Spins with a mean orientation of 0 at $T = 0$ have been excluded to avoid plotting spurious transitions, as discussed in Sec. 4. Inset: Same plot for single unit cell.

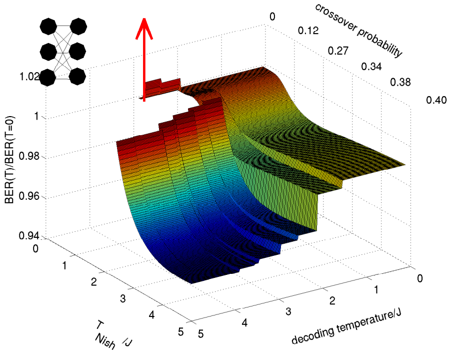

Figure S3: Analytically calculated bit-error-rate for a truncated single unit-cell of the Chimera graph, plotted as a function of the decoding temperature and the Nishimori temperature. The bit-error-rates are normalized with respect to those obtained using maximum likelihood decoding. The graph used for this analysis appears in the upper left corner of the plot. Here BER is shorthand for bit-error-rate.

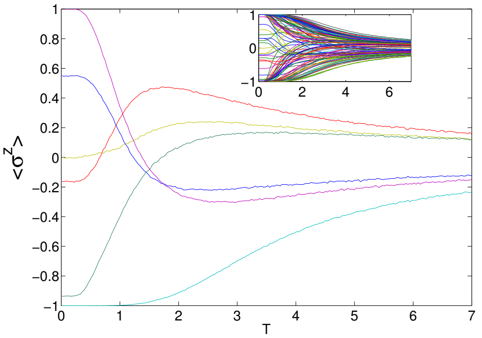

Figure S4: Example of theoretical orientation data for 4x4 chimera unit cell Hamiltonian. Main Figure: selected spins. Inset: All 128.

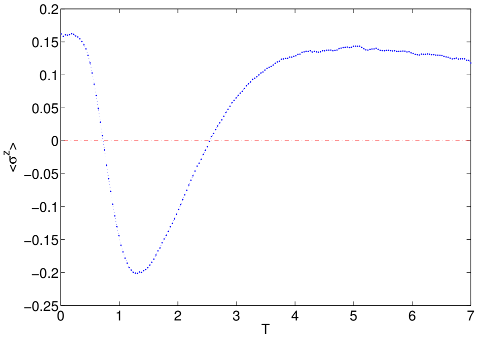

Figure S5: Example of finding spin-sign transitions using linear interpolation.

becomes problematic when the orientation remains very close to zero for a wide range of temperatures when the ground state orientation is zero, as Fig. S6 demonstrates, statistical sampling error leads to this method finding spurious transitions. Because of these spurious transitions these spins are excluded from our analysis.

5 Hamiltonians which Decode Signifcantly Differently than Boltzmann

We have not exhaustively searched for Hamiltonians which give significantly different orientations from the Boltzmann distribution at many different values of temperature, however a few have resulted as byproducts of this project. In the interest of future work, we have included Fig. S6 which displays 4 such Hamiltonians which we have found. Investigation into the cause of these anomalies would be an interesting avenue of future study.

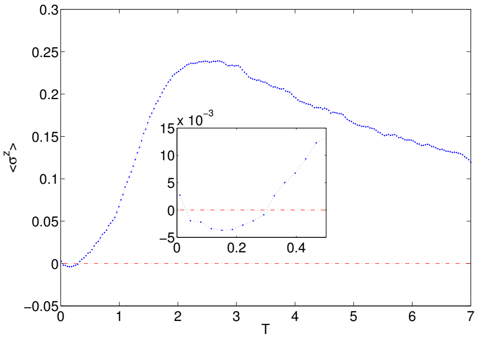

Figure S6: Example of spurious spin-sign transitions found when orientation is zero at $T = 0$, inset is zoom.

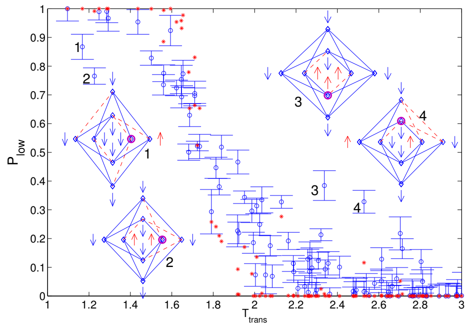

Figure S7: Spins with orientations which differ significantly from Boltzmann distribution predictions. Each Hamiltonian is numbered and the spin which demonstrates the anomalous decoding circled in purple. Dashed lines are anti-ferromagnetic bonds, sold lines are ferro.

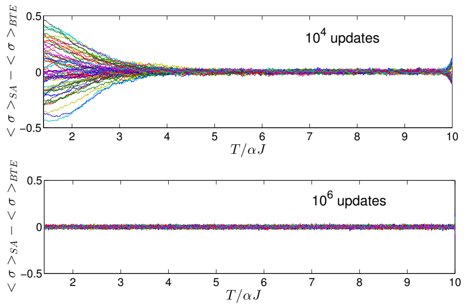

Figure S8: Difference between SA and BTE orientation results for an SA run using a linear sweep from $T/(\alpha J) = 10$ to $T/(\alpha J) = 1.405$. These differences are plotted from all spins with a spin-sign transition within one of the Hamiltonians used to produce Fig. 7 of the main text, chosen at random. With only 10,000 updates the SA data begin to significantly deviate from thermal equilibrium at $T/(\alpha J) \lessapprox 3.5$, while for 1,000,000 updates the data remain in equilibrium down to the temperature of interest, $T/(\alpha J) = 1.405$.

6 Comparison between Simulated Annealing and Bucket Tree Elimination results

While BTE is guaranteed to provide an equilibrium result, it is still interesting to ask whether we can achieve equilibrium though simulated annealing. Fig. S8 demonstrates that for a randomly selected 128 bit Hamiltonian from our data, a linear sweep from $T/(\alpha J) = 10$ to $T/(\alpha J) = 1.405$ can maintain equilibrium (which can be found using BTE) throughout the entire sweep if 1,000,000 updates are used, but fails to maintain equilibrium if only 10,000 are used, as was the case in Fig. 8 of the main paper.

We further examine whether we can qualitatively reproduce the behavior seen in Fig. 8a) of the main paper using SA with many updates as well as control error. As Fig. S9 demonstrates, we can reproduce this qualitative behavior. Slight differences between Fig. S9 and 8a) of the main paper can be attributed either to not all of the Hamiltonians fully equilibrating under SA, or to the fact that restrictions on our available computing power have required us to limit the number of samples used to produce Fig. S9.

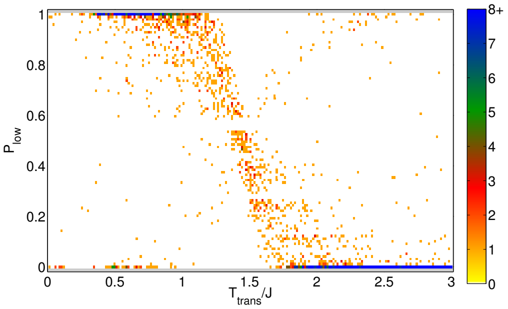

Figure S9: Simulated Annealing data subject to control error. These data were taken with a linear sweep starting from $T/(\alpha J) = 5$ with $5\,10^5$ samples, 5% field control errors and 3% coupler control error. Each randomly generated instance of the error was run for 100 annealing runs and 100 instances were considered for each Hamiltonian.
